# Autoantibody Correlation Signatures in Fibromyalgia and Myalgic Encephalomyelitis/Chronic Fatigue Syndrome: Association with Symptom Severity

**DOI:** 10.3390/biomedicines11020257

**Published:** 2023-01-18

**Authors:** Varvara A. Ryabkova, Natalia Y. Gavrilova, Alina A. Poletaeva, Alexander I. Pukhalenko, Irina A. Koshkina, Leonid P. Churilov, Yehuda Shoenfeld

**Affiliations:** 1 Laboratory of the Mosaic of Autoimmunity, Department of Pathology, Saint Petersburg State University, 199034 Saint Petersburg, Russia; 2 Department of Hospital Therapy Named after Academician M.V. Chernorutskii, Research Institute of Rheumatology and Allergology, Pavlov First Saint Petersburg State Medical University, 197022 Saint Petersburg, Russia; 3 Medical Research Centre “Immunculus”, 105187 Moscow, Russia; 4 LLC “Doctor Koshkina”, 117437 Moscow, Russia; 5 Saint Petersburg Research Institute of Phthisiopulmonology, 191036 Saint Petersburg, Russia; 6 Zabludowicz Center for Autoimmune Diseases, Sheba Medical Center Tel-Hashomer, Ramat-Gan 52621, Israel

**Keywords:** fibromyalgia, myalgic encephalomyelitis/chronic fatigue syndrome, autoantibodies, autoimmunity

## Abstract

Recent studies provide some evidence for the contribution of antibody-mediated autoimmune mechanisms to the nature of fibromyalgia (FM) and myalgic encephalomyelitis/chronic fatigue syndrome (ME/CFS). Much attention was paid to the autoantibodies (AAb) targeting G protein-coupled receptors as natural components of the immune system. However, the natural AAb network is much more extensive, and has not been previously investigated in these disorders. The enzyme immunoassays ELI-Viscero-Test and ELI-Neuro-Test were used to determine changes in serum content of 33 natural AAb to neural, organ-specific and non-tissue-specific autoantigens (a) in 11 ME/CFS patients with comorbid FM; (b) in 11 ME/CFS patients without FM; (c) in 11 healthy controls. Individual AAb profiles and their correlation with some clinical symptoms were analyzed. Both patients with ME/CFS(−)FM and ME/CFS(+)FM were characterized by more frequent and pronounced deviations in the immunoreactivity to GABA-receptors than healthy controls. Although the level of other natural AAb did not differ between study groups, AAb correlation signatures were altered in patients compared to healthy controls. Both in patients and healthy controls the level of natural AAb to various neural and tissue-specific antigens correlated with the severity of fatigue, bodily pain, depression, anxiety, physical and mental health-related quality of life. Notably, widely different correlation patterns were observed between study groups. Findings from this pilot study provide some evidence that the homeostasis of autoimmune relationships, which are possibly a physiological part of our immune system, may be altered in FM and ME/CFS. The correlation of disease-induced perturbations in individual AAb profiles with some clinical symptoms may arise from the immune system’s ability to reflect qualitative and quantitative changes in antigenic composition of the body.

## 1. Introduction

Fibromyalgia (FM) is a common cause of chronic, widespread, musculoskeletal pain with unclear etiopathogenesis. However, it was demonstrated in a recent breakthrough study that IgG from the patients with FM produced painful sensory hypersensitivities by sensitizing peripheral nociceptive afferents in mice [1]. IgG from patients in this study labeled satellite glial cells and neurons in vivo and in vitro, as well as myelinated fiber tracts and a small number of macrophages and endothelial cells in mouse dorsal root ganglia. Furthermore, FM IgG was bound to human dorsal root ganglia. The same author group further showed that a subset of FM patients had elevated levels of anti-spinal ganglia autoantibodies (AAb) (detected in a cell culture assay), which were associated with more severe symptoms [2]. However, a large variation between individual serum samples was observed, suggesting that only a subset of FM patients have autoreactive IgG to spinal ganglia.

Myalgic encephalomyelitis/chronic fatigue syndrome (ME/CFS) is a disabling medical condition characterized by unexplained and persistent post-exertional fatigue, accompanied by a variety of symptoms related to cognitive impairment, immunological, endocrine, and autonomic dysfunction [3]. The common core symptoms of fatigue, sleep problems, and cognitive difficulties lead to significant comorbidity between FM and ME/CFS, but it can vary depending on the diagnostic criteria used [4]. While in one study 34% of 313 patients diagnosed with ME/CFS had comorbid FM according to the American College of Rheumatology (ACR) old diagnostic criteria of 1990, the other research group reported that the prevalence of FM among patients with ME/CFS could reach 50% when ACR 2010 newer diagnostic criteria were applied [4]. The recent meta-analysis confirmed a prominent clinical overlap between FM and ME/CFS [5]. Although the exact pathogenesis of ME/CFS is still unknown, according to the most widely held hypothesis, it is a complex multifactorial syndrome with immunological, metabolic, mitochondrial, autonomic and adrenal dysfunction as the key pathogenetic mechanisms [6,7]. Emerging evidence suggests that disorders of autoimmunity play an important role in postinfectious ME/CFS, and that targeting AAb could be a promising treatment approach [8,9]. 

At the same time, an increased level of any AAb in the serum of a patient is not an ultimate sign of autoimmune disease, but can indicate changes in expression and/or excretion of the corresponding antigens [8]. From this point of view, not only increased, but also decreased levels of AAb are relevant. Recently, a network of natural AAb against adrenergic, muscarinic, and other G-protein coupled receptors (GPCR) has been described, which was shown to be dysregulated in a wide spectrum of diseases (not only autoimmune ones) [10,11]. We assume that a group of AAb to GPCR is only a special case of a complex natural AAb network. The purpose of our study was to compare these networks between patients suffering from ME/CFS, with and without comorbid FM and health controls.

## 2. Materials and Methods

### 2.1. Patients and Controls

Patients were included in the study if they met all three of the most commonly used sets of ME/CFS diagnostic criteria (Fukuda et al. (1994) CFS criteria [12], the Canadian Consensus criteria of ME/CFS (2003) [13], and US Institute of Medicine, now called the National Academy of Medicine (IOM/NAM) criteria (2015) [14]). Patients were assigned to the ME/CFS(+)FM or ME/CFS(−)FM groups depending on whether they met ACR 2016 diagnostic criteria for FM [15]. The third group consisted of apparently healthy controls (HC). Individuals with any autoimmune diseases and those who had any acute illness during the last 3 months were excluded from the study. The study was approved by the Ethics Committee of Saint Petersburg State University. All participants gave informed consent.

### 2.2. Questionnaires for Symptom Scoring

The common ME/CFS and FM symptoms (including depression and anxiety) as well as baseline health status were assessed in both patients and HC using the following instruments: the Short Form 36 Health Survey (SF-36), the Multidimensional Fatigue Inventory (MFI), DePaul Symptom Questionnaire-Short form (DSQ-SF), and Hospital Anxiety and Depression Scale (HADS). The SF-36 includes the following subject-reported evaluations about current health status: physical and social functioning, physical and emotional limitations, vitality, pain, and general and mental health [16]. The MFI comprises a 20-item self-reported questionnaire focused on general, physical and mental fatigue, reduced activity, and reduced motivation [17]. Cognitive symptoms were assessed based on the data from the DSQ-SF questionnaire. In particular, composite score was calculated for the cognitive symptoms by averaging scores for the frequency and severity (ranged from 0 to 4) of two symptoms of cognitive dysfunction from the DSQ-SF questionnaire (“Problems remembering things” and “Difficulty paying attention for a long period of time”). HADS is a reliable scale for identifying and assessing the symptom severity in anxiety disorders and depression, both in patients with somatic diseases and in patients with mental disorders [18]. The score 0–7 for each subscale (depression and anxiety symptoms) indicated “normal results”; 8–10—“borderline results, or doubtful case of anxiety/depression”; 11 or more—“probable case of anxiety/depression”.

### 2.3. Autoantibody Quantification—ELISA

We defined individual normalized levels of AAb against 21 organ-specific and non-organ-specific antigens and 12 neural antigens using standardized ELISA test systems for semi-quantitative serum AAb evaluation (ELI-Viscero-test-24 and ELI-N-test-12 by Medical Research Center “Immunculus”, Moscow, Russia). The antigens used in the test systems are listed in Table 1.

The pooled control serum was a preparation of polyclonal immunoglobulins of the IgG class, synthesized by B-lymphocytes in response to antigenic stimuli that occurred throughout the life of donors. Immunoglobulins in the control serum were obtained from the blood serum of more than 5000 healthy donors, and brought to a concentration close to physiological (16 mg/mL). Thus, pooled control serum contained population-normalized IgG class polyclonal antibodies to each of the studied antigens, and was used as a universal standard for all tested antigens. Depending on the studied antigen, the pooled control serum was diluted to a final concentration, which was derived on the basis of studies of the level of AAb of a large cohort of individual serum samples from healthy donors. The content of AAb to the studied antigens was evaluated in the conventional units of optical density, and compared to their content in a control pool of sera from healthy donors (taken for 100%). Then, the average individual immunoreactivity (AIR) of the studied samples for each of the antigens in comparison with the pooled control serum was calculated according to the formula:

$$\mathrm{AIR}=\frac{\frac{R\left(ag1\right)*100}{R\left(k1\right)}-100+\frac{R\left(ag2\right)*100}{R\left(k2\right)}-100+\cdots +\frac{R\left(agN\right)*100}{R\left(kN\right)}-100}{24}$$

AIR—the average reactivity of an individual patient’s serum to all studied antigens, expressed as a percentage of the average reactivity of the pooled control serum with the same antigens.

R (ag1, 2, N)—reactivity (in units of optical density) of the patient’s serum with studied antigens.

R(k1, k2, N)—reactivity (in units of optical density) of the pooled control serum with studied antigens.

The normal (physiological) levels of individual AIR are restricted by the range from −30% to 0% (or conditional units ) of the control sample AIR.

To construct immunoreactivity profiles, the deviation (as a percentage of the individual AIR) of the patient’s serum reactivity with each of the antigens was calculated using specialized software according to the formula:

$$R\left(dev\right)agN=\left(\frac{R\left(agN\right)*100}{R\left(kN\right)}\right)-100-AIR$$

R(dev)agN—deviation (as a percentage of the AIR) of the patient’s serum reactivity with antigen N.

R (agN)—reactivity (in units of optical density) of the patient’s serum with studied antigens.

R(kN)—reactivity (in units of optical density) of pooled control serum with studied antigens.

The normal (physiological) R(dev)agN for each AAb is restricted by the range from −15% to +10% (or conditional units) from the individual AIR.

### 2.4. Statistical Analysis

Statistical processing was performed with the Statistica 10.0 software package. A chi-squared test was applied to compare the prevalence of non-physiological AAb deviations from the individual AIR between patients and HC. The Mann–Whitney U test was applied to compare mean R(dev)agN values between patients and HC. Spearman correlation analysis was performed in each study group in order to compare AAb correlation signatures between the groups. We used Chord diagrams to visualize the patterns of Spearman’s rank correlation coefficients between AAb. Spearman correlation analysis was also performed to study the relationship between the severity of different symptoms and R(dev)agN values. Differences were considered significant at *p* < 0.05.

## 3. Results

### Subject Characteristics

The study involved 11 patients with ME/CFS+FM, 11 patients with ME/CFS who did not suffer from FM, and 11 healthy controls. Patient characteristics are shown in Table 2.

Patients from ME/CFS(+)FM and ME/CFS(−)FM groups did not differ from controls in terms of gender and age. While BMI in ME/CFS(−)FM group was similar to the BMI in HC, it was significantly lower in ME/CFS(+)FM group. Regarding race and ethnicity, all patients and HC were Caucasian and not Hispanic.

The illness duration varied, with a range of 1 to 35 years and median value of 6.0 years in the ME/CFS(+)FM group and with a range of 2.5 to 35 years and median value of 7.0 years in the ME/CFS(+)FM group. Four patients (33%) with ME/CFS and comorbid FM and eight patients (73%) without comorbid FM reported an infection-triggered onset of disease.

All scales from SF-36 and MFI were significantly different between ME/CFS(+)FM and HC, as well as between ME/CFS(−)FM and HC (Table 3). Both physical and mental component scores (PCS and MCS, respectively) derived from the SF-36 short survey were, as expected, higher in the control group (*p* < 0.05), indicating better health.

It should be noticed that all patients in our study presented with clinically significant post-exertional malaise (according to the DSQ-SF, they had frequency and severity ratings ≥2 in questions number 2 or 3), which has been considered a key symptom of ME/CFS for over three decades [10,11,12].

Depression and anxiety levels were significantly higher in ME/CFS(−)FM and ME/CFS(+)FM groups. The median HADS-D subclase score indicated probable comorbid depression only in the ME/CFS(+)FM group, and the median HADS-A score corresponded to borderline anxiety in both groups of patients.

A significantly higher proportion of patients in ME/CFS(−)FM and ME/CFS(+)FM groups presented with abnormal levels of AAbs against GABA receptors compared to HC (Table 4). At the same time, the proportion of patients with abnormal peaks of any other AAb in groups of patients did not differ significantly from the controls.

In order to assess the extent of abnormalities in the immunoreactivity profiles of patients, we calculated median absolute deviations of the participants’ serum reactivity towards each of the underlying antigens from AIR (median R(dev)agNs) in each of the three study groups, and then compared the obtained values between patients and controls. No significant differences have been observed.

Based on the concept of the antibodiome as a functional and physiological network of AAb, which reflects the exposome and could be disturbed in the disease process [19], we performed a correlation analysis between R(dev)agNs and some clinical characteristics (BMI, age, SF-36 and MFI-20 subscales, depression, anxiety, composite score for cognitive symptom frequency and severity) in each of the three study groups. The significant correlations are shown in Table 5, Table 6 and Table 7.

**Figure 1:**
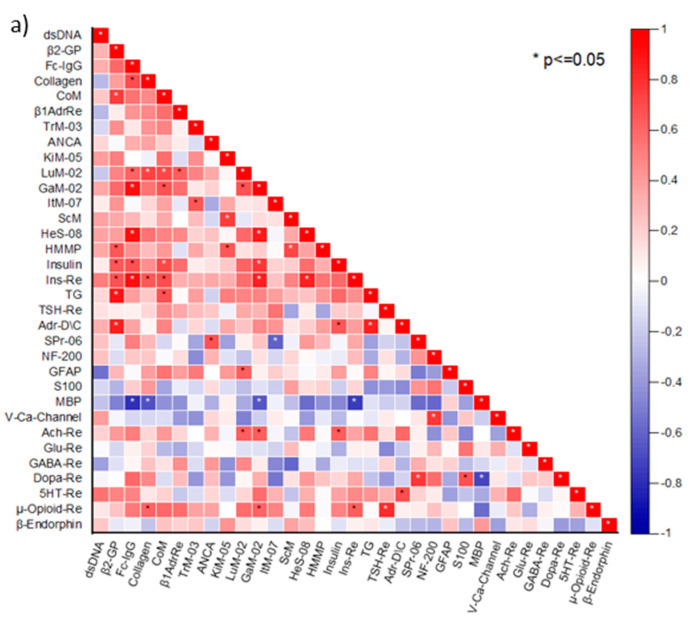

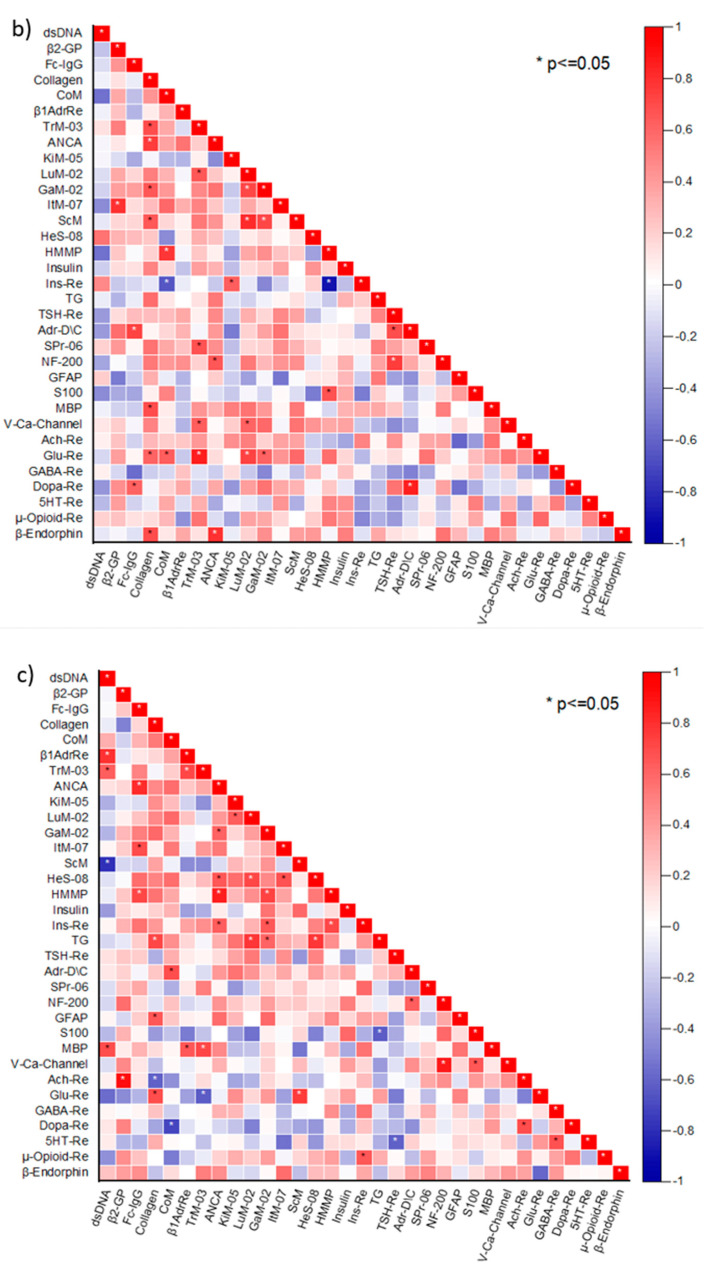
Correlation matrices of AAb targeting organ-specific, neural, and non-organ-specific antigens (denoted by abbreviations as per legend in Table 1) for (**a**) ME/CFS(−)FM (*n* = 11), (**b**) ME/CFS(+)FM (*n* = 11), (**c**) healthy controls (*n* = 11). The color scale bar represents the range of Spearman’s rank correlation coefficient. Significant correlations are marked with *.

To identify changes in the AAb relationships in patients suffering from ME/CFS with and without concomitant FM, we analyzed AAb correlations in the two patient groups separately. A number of correlations between the absolute R(dev)agNs values in both groups were revealed. Disease-specific changes among the studied AAb were identified (Figure 1a,b).

The aforementioned data on the correlation between R(dev)agNs and some symptom scores, even in HC, suggested the presence of physiological relationships among natural AAb. Based on these findings, we expanded our study to include the analysis of HC sera for correlations between the studied AAb (Figure 1c). As previously shown for a few types of AAb (especially those targeting GPCR) [10], we observed that a number of AAb targeting various organ-specific, neural, and non-organ-specific antigens correlated with each other in HC.

**Figure 2:**
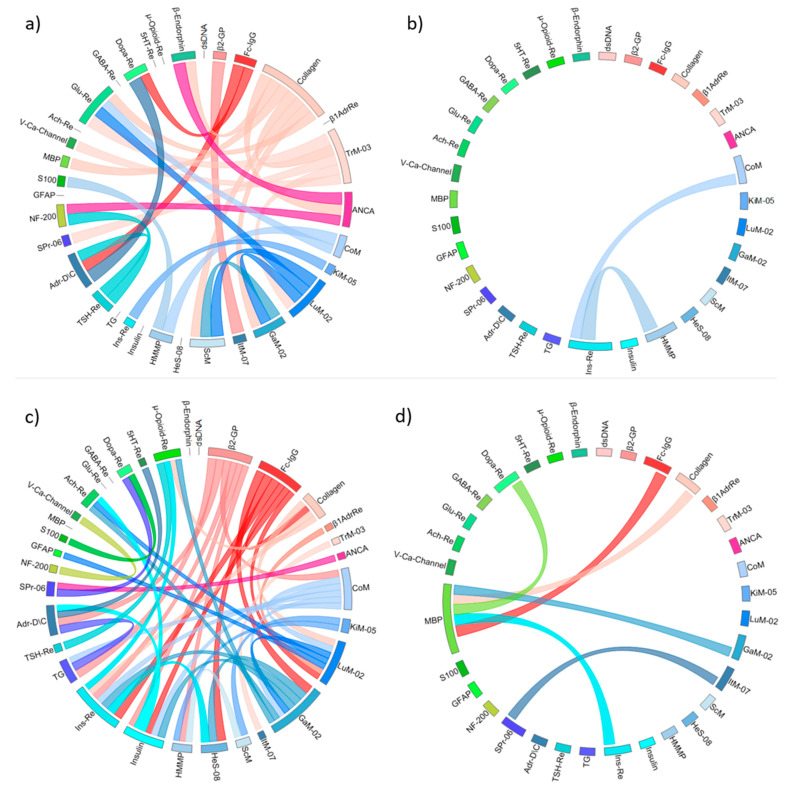

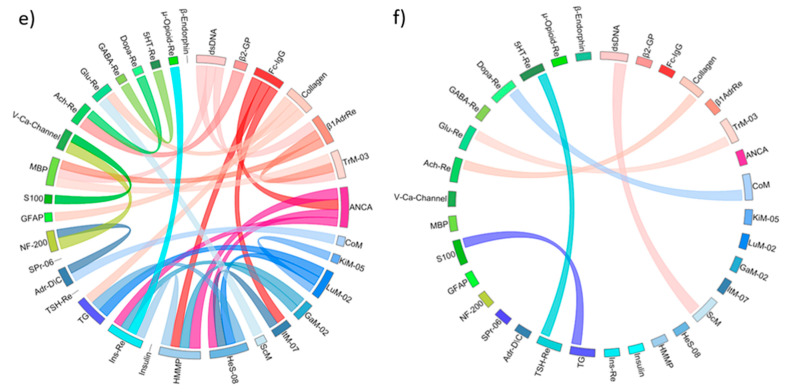
Chord diagrams illustrate the correlation matrices of AAb in the study cohorts. Segments in circles indicate studied AAb (see Table 1 for abbreviations employed), which are grouped according to the target antigen (red/pink = organ-specific, green = neural, blue = non-organ-specific antigens). Chords linking AAb indicate significant correlations (at least *p* < 0.05) according to Spearman rank correlations, while chord thickness is directly proportional to correlation coefficient. For clarity, positive and negative correlations are shown separately. (**a**) Positive correlations, ME/CFS(+)FM; (**b**) Negative correlations, ME/CFS(+)FM; (**c**) Positive correlations, ME/CFS(−)FM; (**d**) Negative correlations, ME/CFS(−)FM; (**e**) Positive correlations, HC; (**f**) Negative correlations, HC.

To gain better insights into the antibodiome, we presented the significant AAb correlations for each study cohort in chord diagrams. The relationship between the Aab from three antigen groups (organ-specific, neural, and non-organ-specific ones), the number of AAb correlations in patients and HC, and the loss of normal correlation signatures in the disease are more descriptive in these plots (Figure 2a–f).

## 4. Discussion

The role of autoimmunity in ME/CFS and FM is widely discussed now, largely due to the emerging data on functional anti-GPCR AAb in ME/CFS [8], and successful passive transfer of FM pain from patients to mice [1]. In this context, it has been emphasized that effort and positive resources should be placed into exploring the role of functional AAb, their trigger factors, and their potential evolutional purpose in conditions characterized mostly by somatic symptoms, rather than objectively identifiable signs [20]. It has been suggested that autonomic nervous system imbalance might be causing the symptoms in these conditions and, in turn, might be triggered by the pathogenic functional AAb [21]. At the same time, it should be kept in mind that the presence of AAb does not imply the presence of an autoimmune condition, as AAb are also recognized in non-autoimmune diseases [22,23]. According to the modern interpretation of the phenomenon of physiological autoimmunity, AAb act as adaptive bioregulators of cell functions and growth along with neurotransmitters and hormones, both in health and disease [24]. Moreover, there is a concept of “Immunculus” or “Immune Homuculus” based on the assumption that the network of physiological autoreactive antiidiotypic AAb may dynamically reflect the whole individual antigenome as a totality of internal immunological images of the autoantigens [25,26,27].

The results obtained in our study suggest that ME/CFS and FM are rather not autoimmune diseases, but conditions with dysregulated natural autoimmunity. In particular, while 54.5% and 45.5% of patients in ME/CFS(+)FM and ME/CFS(−)FM groups presented with abnormal absolute deviations of serum reactivity against GABA Re compared to none in the control group, these changes were not very pronounced (since no significant differences have been observed in median R(dev)agNs between the three groups). Classical autoimmune diseases with pathogenic AAb are characterized with a significant increase in the level of the AAb. While immunological and inflammatory changes have been repeatedly reported in many ME/CFS patients, these changes tend to be most pronounced during the first 3 years of the disease [28]. However, according to the longitudinal studies of varying duration, less than 10% of patients with ME/CFS fully recover [3]. These findings support the hypothesis that not pathogenic AAb, but dysregulated natural autoimmunity is of crucial importance in the pathogenesis of ME/CFS. According to the concept of physiological autoimmunity, quantitative changes in the content of natural AAb are related to variations of expression and secretion of the relative antigen, reflecting functional state of the corresponding cell type [19,27]. GABA Re is an element of endogenous stress-regulating mechanisms preventing distress [29]. The last factor has long been considered as increasing the risk of ME/CFS [30]. An imbalance between excitatory and inhibitory neurotransmission has been linked to ME/CFS and FM [31]. Interestingly, pregabalin, which is one of the three FDA-approved drugs for the treatment of FM, is a lipophilic analogue of GABA. At the same time, it neither acts like GABA nor binds to GABA receptors, but binds strongly to the auxiliary alpha-2 delta subunit of the presynaptic voltage-gated calcium channel receptor to reduce the activation of postsynaptic neurotransmitter release [31]. Regarding some potential substances for future clinical research in ME/CFS and FM, it should be mentioned that a number of complementary dietary supplements have been reported to rebalance glutamate:GABA neurotransmission, namely Omega-3 PUFAs, CoQ10, Withania Somnifera (Ashwagandha, Indian Ginseng), N-acetylcysteine, vitamin B12, curcumin (contained in turmeric), zinc, magnesium, 2-aminoethanesulfonic acid (L-Taurine), and carnitine (L-Carnitine) [32].

The majority of AAb, for which significant correlations with the symptom scores were identified in HC, target neural antigens, which was expected as the analyzed symptoms are neuropsychological. This association was disturbed in both ME/CFS(+)FM and ME/CFS(−)FM groups—the number of AAb targeting internal organ-specific and non-tissue specific antigens correlated with the symptoms scores in these groups. Thus, our findings allow us to assume that ME/CFS and FM are organic multisystem diseases, rather than psychological disorders.

The pattern of correlations found in ME/CFS(−)FM (according to the manufacturer of the ELI-tests) suggests the roles of the gut microbiome, disturbed detoxification mechanisms (namely, liver and kidney functioning), and an inflammatory process in the pelvic organs in the development of symptoms. At the same time, the mechanisms underlying the observed correlations remain largely unclear and can differ between patients and HC. For example, anti-GABA-Re AAb were associated with a lower mental component score, i.e., worse self-perceived mental health in HC, while in patients from the ME/CFS(−)FM group, these AAb were inversely correlated with depression.

Changes in immunoreactivity to β-endorphin were associated with more pronounced fatigue in both ME/CFS(−)FM and ME/CFS(+)FM groups, but not in HC. These findings suggest the dysfunction of the endogenous opioid system in ME/CFS and FM. Interestingly, a significant factor that differentiates β-endorphin from other endogenous opiates is its high affinity for and lasting effect on μ-opioid receptors [33]. When comparing patient groups and HC, with regard to the associations of bodily pain with the studied AAb, it can be noted that patients from both groups abrogated the normal interconnection between anti-μ-Opioid-Re AAb and pain intensity. It was shown by Schrepf et al., in the study employing PET and fMRI together, that dysregulation of the endogenous opiate system in FM could lead to less excitation in antinociceptive brain regions by incoming noxious stimulation [34]. Hyperalgesia and allodynia commonly observed in this population were consistent with the imaging findings. 

Increasing evidence suggests that GFAP might be a biomarker for a number of neurological conditions, which is characterized by strong brain-specificity and high expression level in the brain. Brain injury causes the release of GFAP and its breakdown products from injured astrocytes to the extracellular space, where these proteins equilibrate into the subarachnoid CSF compartment, then release into the circulating blood by the glymphatic pathway or by diffusing past the (possibly compromised) brain blood barrier [35]. It has been also reported that GFAP can serve as an autoantigen, triggering AAb response in a subset of patients [35]. In our study, changes in anti-GFAP AAb level were positively associated with worse mental component score in the ME/CFS(+)FM group and with illness duration in the ME/CFS(−)FM group.

The network-based analysis has been recently implemented in the study of physiological autoimmunity and its disturbances. In particular, distinct signatures of anti-GPCR AAb in HC, which were influenced by age, gender, and various diseases have been revealed [10].

In our study, we determined the correlation signatures of some AAb targeting neural, internal organ-specific and non-organ-specific in HC, and in patients suffering from ME/CFS with and without comorbid FM. AAb to β2-GP, Fc-IgG, CoM, LuM-02, and GaM-02 had obviously more associations with other AAb in the ME/CFS(−)FM group than in HC. Danilenko et al. [36] showed, with the same method as in our study, that anti-β2-GP AAb were increased in ME/CFS patients, but not in healthy participants, which alluded to the link between ME/CFS and antiphospholipid syndrome, earlier suspected by Berg et al. in 1999 [37]. 

The ME/CFS(+)FM group was characterized by an increase in the number of associations of anti-collagen AAb and anti-TrM-03 AAb with other AAb. It has been recently reported that 81% of patients with ME/CFS and/or FM met Brighton criteria for hypermobility syndrome, and 18% met 2017 hypermobile Ehlers–Danlos syndrome criteria. Hypermobility scores significantly predicted symptom levels in these patients [38]. Notably, a high titer of AAb to type I collagen was found in patients with undifferentiated connective tissue dysplasia and joint hypermobility by other group of authors [39]. Earlier, such individuals were also demonstrated to be predisposed to anti-thyroid autoimmunity [40]. Another feature of the AAb correlation pattern in the ME/CFS(+)FM group was the abolished intragroup correlations between anti-neural AAb.

A major limitation encountered in this study was the small sample size. The other limitation was the absence of one universally accepted case definition of ME/CFS. When the underlying pathology of an illness is unknown, as with ME/CFS, there is no gold standard against which to assess the sensitivity or specificity of a case definition [12]; therefore, we had to use three sets of diagnostic criteria to confirm ME/CFS in our patients. We also did not have an opportunity to check the patients for other frequent comorbidities of ME/CFS and FM (e.g., irritable bowel syndrome, Ehlers–Danlos syndrome, postural orthostatic tachycardia syndrome etc.). One could suggest that these comorbidities may also have an impact on the natural AAb patterns. Further research is needed to confirm this suggestion.

Based on our observations, we assume that AAb are natural components of the immune system and may become dysregulated, not only in classical autoimmune conditions, but also in FM and ME/CFS. This assumption is in accordance with the perception of the role of the immune system in homeostatic regulation beyond host defense.

## Figures and Tables

**Table 1:** List of antigens, included in the test systems ELI-Viscero-test-24 and ELI-N-test-12.

No	Antigen	Abbreviation
1	Double stranded deoxyribonucleic acid	dsDNA
2	β2-glycoprotein-I	β2-GP
3	Fc-fragments of IgG	Fc-IgG
4	Collagen type IV	Collagen
5	Membrane antigen of cardiomyocytes	CoM
6	β1-adrenergic receptors of cardiomyocytes	β1AdrRe
7	Platelet membrane antigen	TrM-03
8	Cytoplasmic antigen of neutrophils	ANCA
9	Membrane antigen of renal glomerular cells	KiM-05
10	Membrane antigen of pulmonary alveolocytes	LuM-02
11	Membrane antigen of gastric wall cells	GaM-02
12	Membrane antigen of small intestine wall cells	ItM-07
13	Membrane antigen of colon wall cells	ScM
14	Cytoplasmic antigen of hepatocytes	HeS-08
15	Membrane antigen of hepatocyte mitochondria	HMMP
16	Human insulin	Insulin
17	Insulin receptors	Ins-Re
18	Thyroglobulin	TG
19	Thyrotropin receptor	TSH-Re
20	Membrane antigen of adrenal medulla cells	Adr-D\C
21	Membrane antigen of sperm and prostate cells	SPr-06
22	Neurofilament protein 200	NF-200
23	Glial fibrillary acidic protein	GFAP
24	S100 protein	S100
25	Myelin basic protein	MBP
26	Voltage-dependent calcium channel	V-Ca-Channel
27	N-cholinergic receptors	Ach-Re
28	Glutamate receptors	Glu-Re
29	γ-aminobutyric acid receptors	GABA-Re
30	Dopamine receptors	Dopa-Re
31	Serotonin receptors	5HT-Re
32	μ-opioid receptors	μ-Opioid-Re
33	β-endorphin	β-Endorphin

**Table 2:** Study population characteristics. BMI—body mass index, FM—fibromyalgia, HC—heathy controls, IQR—interquartile range, ME/CFS—myalgic encephalomyelitis/chronic fatigue syndrome.

Subject Characteristics		ME/CFS(+)FM (*n* = 11)	ME/CFS(−)FM (*n* = 11)	HC (*n* = 11)	*p*-Values ^1^	
					ME/CFS(+)FM vs. HC	ME/CFS(−)FM vs. HC
Gender	Female	8	9	8	1.000	0.655
	Male	3	2	3		
Age	Median (IQR)	38.0 (31.0–47.0)	30.0 (27.0–45.0)	33.0 (27.0–49.0) 1	0.519	0.562
BMI	Median (IQR)	18.8 (17.9–19.6)	23.8 (19.8–32.3)	21.4 (19.3–25.4)	0.010	0.193
Illness Duration (years)	Median (IQR)	6.0 (3.0–21.5)	7.0 (6.0–10.5)	N/A	N/A	
Onset of disease	Infection-triggered	4	8	N/A	N/A	
	Severe stress(-es)-triggered	5	2			

1—For categorial variable, p-values were derived from Chi-squared test; for continuous variables, p-values were derived from Mann–Whitney U test.

**Table 3:** Fatigue, depression, anxiety, and baseline health status assessment in patients and healthy controls. FM—fibromyalgia, HADS—Hospital Anxiety and Depression Scale, HC—heathy controls, IQR—interquartile range, PCS—physical component score; MCS—mental component score, ME/CFS—myalgic encephalomyelitis/chronic fatigue syndrome, MFI—multidimensional Fatigue Inventory.

Subject Characteristics		ME/CFS(+)FM (*n* = 11)	ME/CFS(−)FM (*n* = 11)	HC (*n* = 11)	*p*-Values	
					ME/CFS(+)FM vs. HC	ME/CFS(−) FM vs. HC
SF-36 Scales Median (IQR)	Physical Functioning	40.0 (30.0–55.0)	45.0 (30.0–75.0)	100.0 (95.0–100.0)	0.000	0.000
	Role physical	0.0 (0.0–0.0)	0.0 (0.0–0.0)	100.0 (50.0–100.0)	0.000	0.000
	Bodily pain	41.0 (31.0–41.0)	74.0 (22.0–100.0)	100.0 (84.0–100.0)	0.000	0.028
	General Health	35.0 (20.0–45.0)	30.0 (25.0–40.0)	87.0 (62.0–92.0)	0.000	0.000
	Vitality	10.0 (0.0–25.0)	10.0 (0.0–20.0)	80.0 (55.0–85.0)	0.000	0.000
	Social functioning	25.0 (0.0–50.0)	25.0 (0.0–37.5)	100.0 (75.0–100.0)	0.000	0.000
	Role emotional	33.3 (0.0–100.0)	0.0 (0.0–100.0)	100.0 (66.7–100.0)	0.040	0.028
	Mental health	52.0 (28.0–56.0)	40.0 (28.0–56.0)	68.0 (52.0–80.0)	0.002	0.001
	PCS	30.6 (26.3–35.0)	35.1 (30.0–45.5)	57.2. (50.8–58.7)	0.000	0.000
	MCS	34.5 (21.1–43.3)	26.0 (21.9–37.0)	53.1 (44.5–55.1)	0.001	0.000
MFI Scales Median (IQR)	General Fatigue	19.0 (18.0–20.0)	19.0 (19.0–20.0)	7.0 (6.0–9.0) 1	0.000	0.000
	Mental Fatigue	16.0 (13.0–18.0)	14.0 (9.0–15.0)	6.0 (5.0–10.0)	0.000	0.010
	Physical Fatigue	16.0 (15.0–20.0)	18.0 (16.0–20.0)	6.0 (5.0–9.0)	0.000	0.000
	Reduced Activity	19.0 (16.0–20.0)	19.0 (17.0–20.0)	9.0 (5.0–11.0)	0.000	0.000
	Reduced Motivation	14.0 (9.0–17.0)	13.0 (11.0–14.0)	9.0 (5.0–11.0)	0.003	0.001
HADS Median (IQR)	Depression subscale	13.0 (10.0–15.0)	11.0 (9.0–16.0)	3.0 (0.0–4.0)	0.000	0.000
	Anxiety subscale	10.0 (7.0–14.0)	10.0 (6.0–12.0)	4.0 (1.0–5.0)	0.001	0.003

**Table 4:** Number of patients and HC with abnormal deviation (as a percentage of the AIR) of the patient’s serum reactivity towards the studied antigens. FM—fibromyalgia, HC—heathy controls, ME/CFS—myalgic encephalomyelitis/chronic fatigue syndrome.

Autoantibodies	ME/CFS(+)FM (*n* = 11)		ME/CFS(−)FM (*n* = 11)		HC (*n* = 11)		*p*-Values	
							ME/CFS(+)FM vs. HC	ME/CFS(−) FM vs. HC
Anti-dsDNA AAb	2	(18.2%)	3	(27.3%)	2	(18.2%)	1.00	0.66
Anti-β2-GP AAb	0	(0.0%)	3	(27.3%)	1	(9.1%)	1.00	0.57
Anti-Fc-IgG AAb	1	(9.1%)	1	(9.1%)	2	(18.2%)	0.61	0.61
Anti-Collagen AAb	4	(36.4%)	1	(9.1%)	3	(27.3%)	0.68	0.34
Anti-CoM AAb	1	(9.1%)	2	(18.2%)	2	(18.2%)	0.61	1.00
Anti β1 Adr Re AAb	2	(18.2%)	2	(18.2%)	3	(27.3%)	1.00	0.66
Anti TrM-03 AAb	1	(9.1%)	0	(0.0%)	3	(27.3%)	0.34	0.11
Anti-ANCA AAb	1	(9.1%)	2	(18.2%)	2	(18.2%)	0.61	1.00
Anti-KiM-05 AAb	1	(9.1%)	1	(9.1%)	1	(9.1%)	1.00	1.00
Anti-LuM-02 AAb	4	(36.4%)	5	(45.5%)	6	(54.5%)	0.43	0.70
Anti-GaM-02 AAb	1	(9.1%)	3	(27.3%)	2	(18.2%)	0.61	0.66
Anti-ItM-07 AAb	5	(45.5%)	3	(27.3%)	5	(45.5%)	1.00	0.42
Anti-ScM AAb	4	(36.4%)	3	(27.3%)	1	(9.1%)	0.17	0.34
Anti-HeS-08 AAb	0	(0.0%)	4	(36.4%	1	(9.1%)	1.00	0.53
Anti-HMMP AAb	1	(9.1%)	0	(0.0%)	2	(18.2%)	1.00	0.24
Anti-Insulin AAb	1	(9.1%)	2	(18.2%)	1	(9.1%)	1.00	0.61
Anti-Ins-Re AAb	1	(9.1%)	0	(0.0%)	1	(9.1%)	1.00	1.00
Anti-TG AAb	0	(0.0%)	3	(27.3%)	0	(0.0%)	1.00	0.11
Anti-TSH-Re AAb	1	(9.1%)	1	(9.1%)	1	(9.1%)	1.00	1.11
Anti-Adr-D\C AAb	1	(9.1%)	3	(27.3%)	2	(18.2%)	0.61	0.66
Anti-SPr-06 AAb	2	(18.2%)	4	(36.4%)	3	(27.3%)	0.66	0.68
Anti-NF-200 AAb	0	(0.0%)	1	(9.1%)	1	(9.1%)	1.00	1.00
Anti-GFAP AAb	1	(9.1%)	0	(0.0%)	0	(0.0%)	1.00	1.00
Anti-S100 AAb	1	(9.1%)	1	(9.1%)	1	(9.1%)	1.00	1.00
Anti-MBP AAb	0	(0.0%)	0	(0.0%)	2	(18.2%)	1.00	0.48
Anti-V-Ca-Channel AAb	0	(0.0%)	1	(9.1%)	1	(9.1%)	1.00	1.00
Anti-Ach-Re AAb	0	(0.0%)	1	(9.1%)	0	(0.0%)	1.00	1.00
Anti-Glu-Re AAb	1	(9.1%)	0	(0.0%)	2	(18.2%)	1.00	0.48
Anti-GABA-Re AAb	6	(54.5%)	5	(45.5%)	0	(0.0%)	0.0	0.06
Anti-Dopa-Re AAb	2	(18.2%)	2	(18.2%)	2	(18.2%)	1.00	1.00
Anti-5HT-Re AAb	1	(9.1%)	1	(9.1%)	1	(9.1%)	1.00	1.00
Anti-μ-Opioid-Re AAb	1	(9.1%)	2	(18.2%)	1	(9.1%)	1.00	0.61
Anti-β-Endorphin AAb	1	(9.1%)	3	(27.3%)	2	(18.2%)	0.61	0.66

**Table 5:** Clinical correlations of the absolute AAb deviations from AIR in the group of patients suffering from ME/CFS without comorbid FM. (Spearman correlation coefficient r and *p*-value).

Subject Characteristics		Autoantibodies	r	*p*
SF-36 Scales Median (IQR)	Bodily pain	Anti-KiM-05 AAb	+0.611	0.046
		Anti-ScM AAb	+0.752	0.008
		Anti-HMMP AAb	+0.795	0.003
	Physical component score	Anti-ItM-07 AAb	+0.724	0.012
	Mental component score	No significant correlation was found		
MFI Scales Median (IQR)	General Fatigue	Anti-ItM-07 AAb	−0.659	0.027
		Anti-SPr-06 AAb	+0.697	0.017
	Mental Fatigue	No significant correlation was found		
	Physical Fatigue	No significant correlation was found		
	Reduced Activity	Anti-V-Ca-Channel AAb	−0.620	0.042
		Anti-Ach-Re AAb	+0.623	0.041
	Reduced Motivation	Anti-β Endorphin AAb	+0.665	0.026
HADS	Depression subscale	Anti-GABA-Re AAb	−0.639	0.034
	Anxiety subscale	Anti-SPr-06 AAb	−0.639	0.034
		Anti-Dopa-Re AAb	−0.762	0.006
		Anti-MBP AAb	0.820	0.002
DSQ-SF	Composite score of cognitive symptoms	No significant correlation was found		
	Illness duration	Anti-Dopa-Re AAb	−0.652	0.03
		Anti-GFAP AAb	+0.668	0.025
		Anti-TG AAb	+0.728	0.011
	Age	Anti-Fc-IgG AAb	−0.741	0.009
		Anti-GaM-02 AAb	−0.636	0.035
		Anti-HeS-08 AAb	−0.800	0.003
	BMI	No significant correlation was found		

**Table 6:** Clinical correlations of the absolute AAb deviations from AIR in the group of patients suffering from ME/CFS with comorbid FM. (Spearman correlation coefficient r and *p*-value).

Subject Characteristics		Autoantibodies	r	*p*
SF-36 Scales Median (IQR)	Bodily pain	Anti-CoM AAb	+0.685	0.04
	Physical component score	No significant correlation was found		
	Mental component score	Anti-GFAP AAb	−0.774	0.005
		Anti-ItM-07 AAb	+0.612	0.046
	General fatigue	Anti-V-Ca-Channel AAb	+0.675	0.023
	Mental Fatigue	Anti-β Endorphin AAb	+0.773	0.005
	Physical Fatigue	Anti-V-Ca-Channel AAb	+0.646	0.032
		Anti-TG AAb	−0.785	0.004
	Reduced Activity	Anti-TG AAb	−0.613	0.045
		Anti-SPr-06 AAb	−0.832	0.001
		Anti-β Endorphin AAb	+0.629	0.038
	Reduced Motivation	Anti-5HT-Re AAb	−0.772	0.005
HADS	Depression subscale	Anti- β2-GP AAb	−0.629	0.038
		Anti-ItM-07 AAb	−0.627	0.039
	Anxiety subscale	Anti-dsDNA AAb	+0.700	0.017
DSQ-SF	Composite score of cognitive symptoms	Anti-MBP AAb	+0.617	0.043
	Illness duration	No significant correlation was found		
Age		No significant correlation was found		
BMI		Anti-dsDNA AAb	+0.662	0.026

**Table 7:** Clinical correlations of the absolute AAb deviations from AIR in HC group. (Spearman correlation coefficient r and *p*-value).

Subject Characteristics		Autoantibodies	r	*p*
SF-36 Scales Median (IQR)	Bodily pain	Anti-CoM AAb	+0.774	0.005
		Anti-μ-Opioid-Re AAb	−0.719	0.013
	Physical component score	Anti-CoM AAb	+0.636	0.035
	Mental component score	Anti-Dopa-Re AAb	−0.637	0.035
		Anti-GABA-Re AAb	−0.664	0.026
MFI ScalesMedian (IQR)	General Fatigue	No significant correlation was found		
	Mental fatigue	Anti-CoM AAb	−0.647	0.031
	Physical Fatigue	No significant correlation was found		
	Reduced Activity	Anti-Adr-D\C AAb	−0.644	0.033
	Reduced Motivation	No significant correlation was found		
HADS	Depression subscale	Anti-LuM-02 AAb	−0.617	0.043
		Anti-Adr-D\C AAb	−0.683	0.020
	Anxiety subscale	Anti-μ-Opioid-Re AAb	+0.817	0.002
		Anti-Ach-Re AAb	+0.696	0.017
		Anti-Dopa-Re AAb	+0.646	0.032
DSQ-SF	Composite score of cognitive symptoms	Anti-μ-Opioid-Re AAb	+0.861	0.001
Age		Anti-Ins-Re AAb	−0.657	0.028
BMI		Anti-CoM AAb	−0.737	0.010
		Anti-ItM-07 AAb	−0.618	0.043
		Anti-HeS-08 AAb	−0.706	0.015
		Anti-TG AAb	−0.679	0.022
		Anti-S100 AAb	+0.632	0.037

## Data Availability

Not applicable.
